# Medial Open-Wedge Supramalleolar Osteotomy for Patients with Takakura 3B Ankle Osteoarthritis: A Mid- to Long-Term Study

**DOI:** 10.1155/2019/7630868

**Published:** 2019-06-17

**Authors:** Yang Xu, Xiang-yang Xu

**Affiliations:** ^1^Ruijin Hospital North, Shanghai Jiao Tong University School of Medicine, Shanghai, China; ^2^Ruijin Hospital, Shanghai Jiao Tong University School of Medicine, Shanghai, China

## Abstract

It is controversial whether supramalleolar osteotomy is suitable for Takakura Stage 3B osteoarthritis or not. The aim of this study was to evaluate the outcomes of supramalleolar osteotomy in patients with Takakura 3B osteoarthritis. From February 2008 to August 2013, supramalleolar osteotomy was performed in 21 patients matching the inclusion criteria. The mean patient age at operation was 53.7±5.8 years (range: 39 to 61 years). The mean duration of follow-up was 87.7±19.5 months (range: 61 to 125 months). The radiologic evaluation included the tibial articular surface (TAS) angle, tibial lateral surface (TLS) angle, and talar tilt (TT) angle. Functional assessment was performed with use of the AOFAS, VAS, SF-36, and AOS. All patients were followed. TAS angle improved from 82.8±2.4 to 90.3±2.3. TLS angle changed from 77.5±2.8 to 79.4±2.7. The preoperative TT angle and postoperative TT angle were 13.4±3.6 to 4.8±3.6, respectively. For functional evaluation, the preoperative VAS and AOFAS-AH scores were 5.7±1.3 and 48.0±15.8, while the postoperative VAS and AOFAS-AH scores were 2.5±1.9 and 74.8±11.5. The mean SF-36 scale improved from 41.2±13.1 to 66.7±14.9. The AOS score improved from 61.4±12.5 to 27.5±17.8. 1 patient underwent total ankle replacement 3 years postoperatively. 4 patients remained stage 3B including the TAR one. 4 improved to stage 3A, 11 improved to stage 2, and 2 improved to stage 1. Supramalleolar osteotomy combined with auxiliary procedures can restore the malalignment of ankle joint and modify the abnormal stress distribution so as to achieve functional improvement and improve radiographic stages.

## 1. Introduction

According to Takakura et al., varus ankle arthritis is classified into four stages in frontal view weight-bearing radiographs of ankle joint: 1 early sclerosis and formation of osteophytes without changing of ankle joint space; 2 narrowing of medial joint space without subchondral bone contact; 3 obliteration of ankle space with subchondral bone contact; 4 varus ankle joint with complete bone contact. And Stage 3 is divided into 3A ankle joint obliteration limited to the medial malleolus and 3B ankle joint obliteration was extended to the roof of the dome of talus [1, 2]. Supramalleolar osteotomy is one of the joint-preserving methods for the treatment of varus type ankle arthritis, whereas the indications of this procedure are still controversial.

Joint-preserving procedures are still very important because many patients do not want to sacrifice the native joints. For varus type ankle osteoarthritis, supramalleolar osteotomy could restore the normal alignment of ankle and preserve the native ankle joint, which is particularly important for young patients with severe varus ankle osteoarthritis. Although supramalleolar osteotomy has been reported to result in substantial functional improvement and malalignment and achieve good outcomes, it was often recommended for early to midstage ankle arthritis (stage 2 or stage 3A) [1, 3–8]. But in our clinical practice, by performing supramalleolar osteotomy for stage 3B ankle arthritis we did notice positive postoperative results.

This study hypothesized that supramalleolar osteotomy combined with auxiliary procedures such as calcaneal osteotomy and lateral ligament reconstruction are effective for the treatment of Takakura 3B ankle arthritis. It can restore the malalignment of ankle joint and modify the abnormal stress distribution so as to achieve functional improvement and reverse radiographic stages. The purpose of this study was to evaluate the radiographic and functional outcomes of supramalleolar osteotomy combined with auxiliary procedures for stage 3B varus type ankle osteoarthritis.

## 2. Patients and Methods

This retrospective study was approved by institutional review board. From February 2008 to August 2013, supramalleolar osteotomy was performed in 21 patients who had symptomatic Takakura 3B ankle arthritis matching the inclusion criteria, including 3 men and 18 women. The mean patient age at operation was 53.7±5.8 years (range: 39 to 61 years). The mean duration of follow-up was 87.7±19.5 months (range: 61 to 125 months).

The inclusion criteria were (1) Takakura stage 3B ankle arthritis; (2) with clinical symptoms, such as walking pain and limitation of daily activities; (3) patients with follow-up time more than 5 years; (4) regional cartilage destruction on the medial side with more than 50% remaining cartilage on the lateral side in preoperative MRI scans.

The exclusion criteria were (1) end-stage ankle arthritis with global severe cartilage destruction on preoperative MRI scans and stage 1, 2, or 3A ankle arthritis; (2) patients with neuropathic arthropathy or rheumatoid arthritis; (3) patients who had severe osteoporosis or large bone loss around ankle joint; (4) patients who had ankle surgery other than supramalleolar osteotomy like ankle joint distraction or osteotomy around ankle joint.

**Figure 1:**
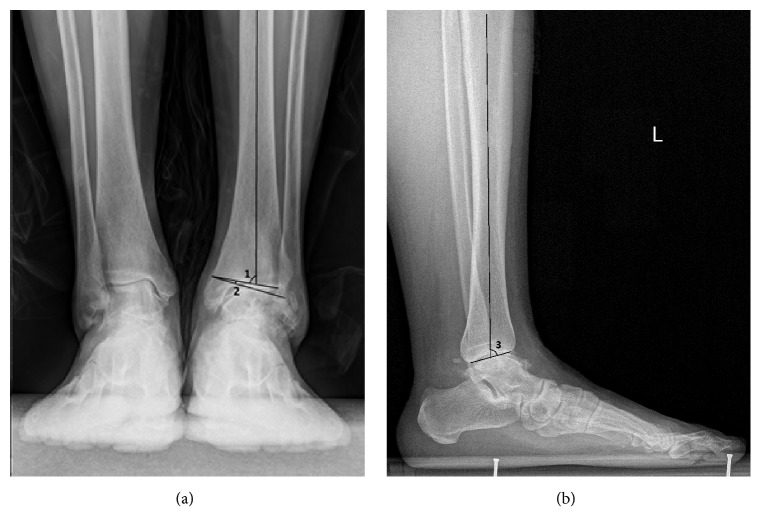
(a) 1: TAS: tibial articular surface angle, 2: TT: talar tilt angle. (b) 3: TLS: tibial lateral surface angle.

Standard weight-bearing radiographs of ankle joints were performed preoperatively, including anteroposterior (AP) and lateral views. The study also observed hindfoot varus deformity. It was recorded via hindfoot alignment view, which was described by Saltzman and el-Khoury [9]. In this study, preoperative MRI was also performed for each patient to see the damage situation of ankle joint cartilage. Supramalleolar osteotomy was performed for 3B ankle arthritis with regional cartilage destruction on the medial side with more than 50% remaining cartilage on the lateral side. The radiologic evaluation included the tibial articular surface (TAS) angle, tibial lateral surface (TLS) angle, and talar tilt (TT) angle. In this study, the postoperative Takakura 1 ankle osteoarthritis was defined as TT angle ≤ 1° without narrowing of ankle joint space (Figures 1(a) and 1(b)).

Functional assessment was performed with use of the American Orthopaedic Foot and Ankle Society ankle-hindfoot (AOFAS-AH) scale, the Visual Analogue Scale (VAS), the Short Form-36 (SF-36) scale, and the Ankle Osteoarthritis scale (AOS). Satisfactory results in this study were regarded as improvements to other stages, which were often accompanied with pain relief [10]. We think single scale especially AOFAS score cannot reflect the real situation of patients. So we combined several scales to assess the function of ankle joint.

### 2.1. Surgical Technique

A debridement of osteophytes around ankle joint was performed and soft tissue was released thoroughly especially the medial ligament first. The medial ligament was performed through a medial longitudinal approach. It was cut like a “Z” shape to make sure the talus could return to normal site. The supramalleolar osteotomy was performed through a medial longitudinal approach. A K-wire was placed into the distal tibial about 4-5 cm above the ankle mortise to guide the osteotomy. The osteotomy was parallel to the tibial plafond. The study used structural allograft to fulfill the supramalleolar osteotomy. Intraoperative visualization and fluoroscopy were used to assess the adequacy of correction of distal tibial varus deformity. The study aimed to make TAS angle in neutral position or a little overcorrected but TAS angle was no more than 95°(90°-95°) [11]. Then the osteotomy was fixed with a plate and interlocking screws. The foot was pushed intraoperatively to simulate weight-bearing situation to see whether the released talus could return to normal. In this position, the medial ligament was sutured. If there was laxity of lateral ligament, an augment procedure or reconstruction procedure was performed [12, 13].

The correction was assessed with the use of fluoroscopy. If it still remained residual hindfoot deformity after supramalleolar osteotomy, then the calcaneal osteotomy was performed. For patients had residual hindfoot deformity, a calcaneal lateral sliding osteotomy was performed to keep hindfoot a little valgus (<5°).

### 2.2. Postoperative Management

A cast was used for 6 weeks. Part weight bearing was allowed 6 weeks after operation with the cast. Full weight bearing was allowed two to three months after operation. Patients were required to go to outpatient at 6 and 8 weeks after operation and take weight-bearing radiographs. Patients came to outpatient 3 months, 6 months, and 1 year after operation to estimate the function of ankle. Then, patients came to hospital annually to take radiographs and estimate the function of ankle joints.

### 2.3. Statistical Analysis

All analyses were performed with SAS software version 8.1 (SAS Institute Inc, Cary, North Carolina). Descriptive statistics were calculated as mean± standard deviation. The paired Student t test was adopted to compare the preoperative and postoperative radiographic measurements and AOFAS-AH, SF-36, AOS, and VAS scores. The significance level was set at P<0.05.

## 3. Results

There was no loss of follow-up. Among the 21 patients, 9 patients underwent calcaneal osteotomies and 12 underwent lateral ligament reconstructions (2 using autograft and 10 using allograft). Modified Broström procedure was performed in 1 patient. Only 1 patient underwent total ankle arthroplasty 3 years after operation because of persistent pain. The mean BMI was 25.0±3.2. 18 patients had a history of ankle sprain. The mean duration of symptom was 18.8±13.6 years (Tables 1 and 2).

TAS angle improved from 82.8±2.4 to 90.3±2.3 (p<0.001). TLS angle changed from 77.5±2.8 to 79.4±2.7 (p=0.0047). The preoperative TT angle and postoperative TT angle were 13.4±3.6 to 4.8±3.6, respectively (p<0.001). For functional evaluation, the preoperative VAS and AOFAS-AH scores were 5.7±1.3 and 48.0±15.8, while the postoperative VAS and AOFAS-AH scores were 2.5±1.9 and 74.8±11.5 (p<0.001). The mean SF-36 scale improved from 41.2±13.1 to 66.7±14.9 (p<0.001). The AOS score improved from 61.4±12.5 to 27.5±17.8 (P<0.001).

**Figure 2:**
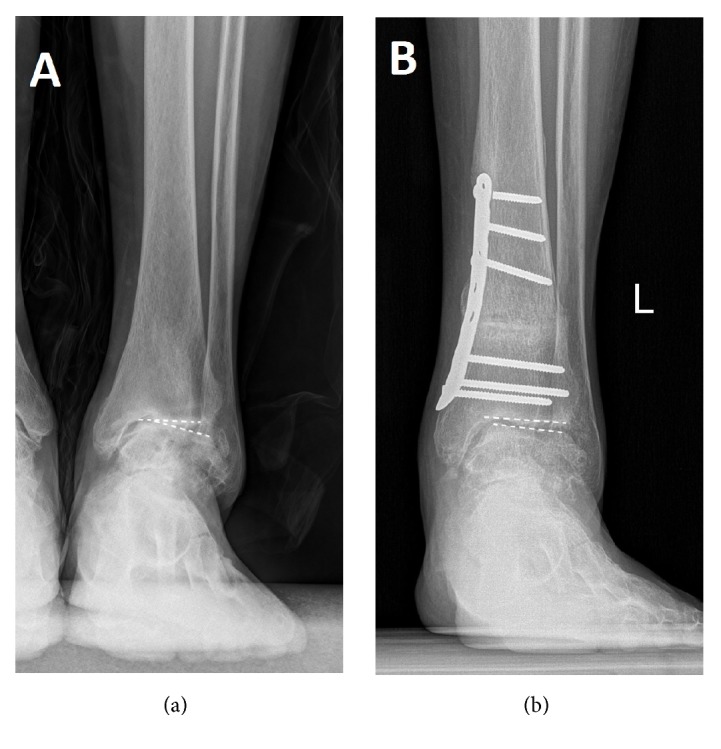
A 46-year-old woman with ankle arthritis before (a) and after (b) the supramalleolar osteotomy, the osteoarthritis stage improved to stage II.

**Figure 3:**
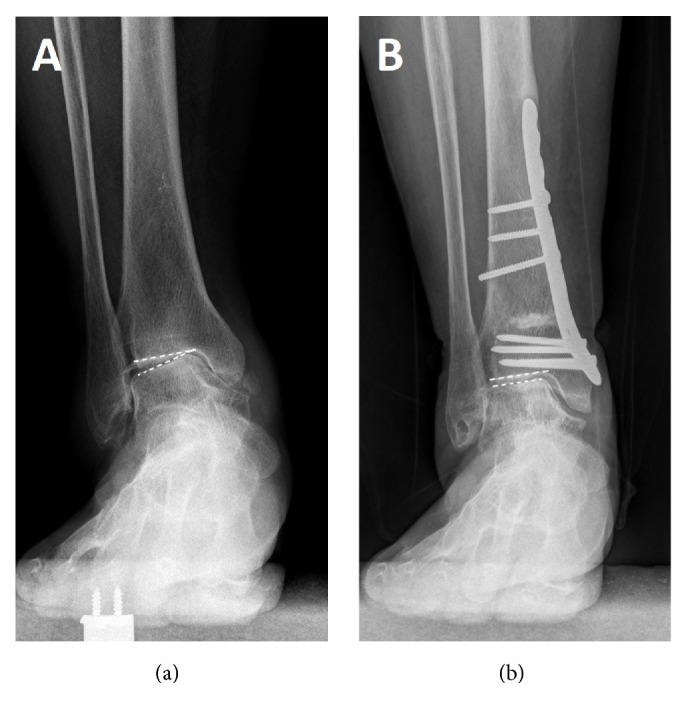
A 56-year-old woman with varus ankle arthritis before (a) and after (b) the supramalleolar osteotomy, the osteoarthritis stage improved to stage I.

**Figure 4:**
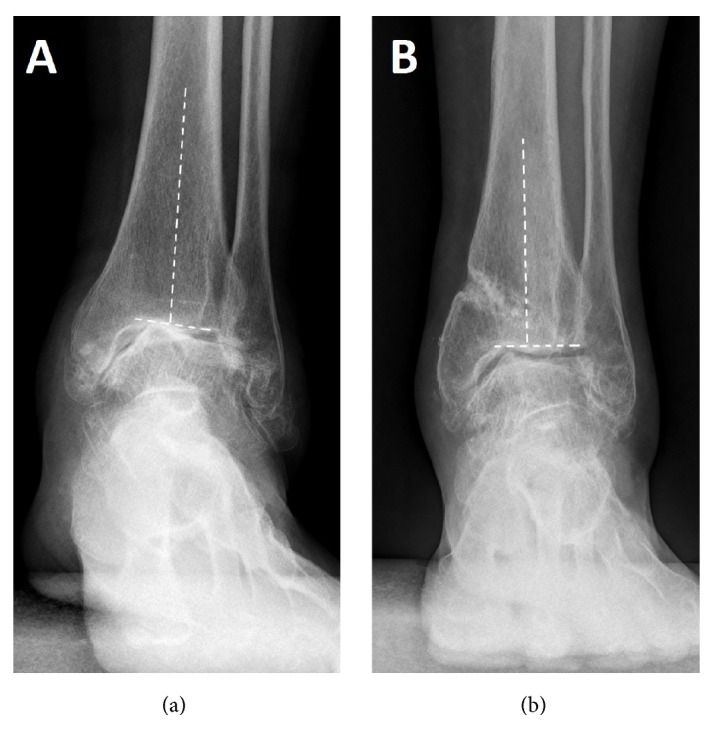
The TAS angle changed before (a) and after (b) supramalleolar osteotomy.

In this study, union was achieved in all patients. 4 patients remained stage 3B including the TAR one. 4 improved to stage 3A, 11 improved to stage 2, and 2 improved to stage 1. (Figures 2, 3, and 4)

## 4. Discussion

For asymmetric varus type ankle arthritis, supramalleolar tibial osteotomy is an effective method. As stated above, it was usually adopted for early to midstage ankle osteoarthritis. Tanaka et al. concluded that supramalleolar osteotomy was not suitable for stage 3B ankle arthritis, whereas Lee et al. thought that supramalleolar osteotomy was effective for stage 3B arthritis. His study included 16 patients, and all 3 cases with 3B ankle arthritis improved to stage 2 [2, 10]. In our opinion, as Haraguchi et al. wrote, the osteoarthritis stage would not adequately reflect the clinical outcomes [14]. In this study, however, most patients with stage 3B varus ankle osteoarthritis achieved pain relief and better radiological manifestations. This study proved that, with proper additional procedures, supramalleolar osteotomy is an effective method for Takakura 3B ankle osteoarthritis.

The cause of varus ankle arthritis was unclear. Tanaka et al. thought it was due to lifestyle, in which people sit cross-legged or with legs tucked under the body [2]. In this study, many patients with varus ankle deformity did not have an ankle fracture history, but most of them (85.7%, 18/21) recalled a history of old ankle sprain. Maybe it was because the weak lateral ligament resulted in varus and internal rotated dislocation of talus. With a long history of impingement at medial ankle joint and abnormal stress distribution, the varus supramalleolar deformity formed.

Knupp et al. thought that most patients with ankle arthritis present with a malaligned hinfoot [15]. Correction of hindfoot axis not only helps normalize the ankle joint load distribution but also prepares for a second surgery like ankle arthrodesis or TAR [15]. This study noticed that, with a long history of supramalleolar deformity and varus talus, many patients presented concomitant hindfoot deformity. So, in this study, near half of patients underwent calcaneal osteotomy. Patients with calcaneal osteotomy did not show any benefit compared with those without calcaneal osteotomy. The purpose of calcaneal osteotomy is to achieve normal alignment of ankle and hindfoot, which is very important for pain relief.

Tanaka thought that a 96° to 98° TAS angle gave better outcomes while Hintermann and Knupp recommended the TAS angle to be a little overcorrection of 92° to 95° [2, 16, 17]. Pagenstert et al. overcorrected the TAS angle to be 90°-95 and achieved positive outcomes [11]. Hintermann et al. corrected the TAS angle to be 2°-4° valgus when the patient had medial cartilage loss [18]. In this study, the TAS angle was made in neutral position or a little overcorrected but no more than 95°. When facing varus ankle arthritis with large TT angle, soft tissue release is very important for it to be reduced and corrected. We do not want to achieve smaller TT angle by overcorrection of TAS.

Lee et al. and Tanaka et al. thought that when talar tilt angle was larger than 7° or 10°, it was difficult to attain a normal ankle joint [2, 4, 10, 19]. However, in our study, most patients with preoperative TT angle larger than 10° achieved pain relief and change of radiographic outcomes. In this study, we performed debridement of osteophytes around ankle joint and release the soft tissue thoroughly especially the medial ligament first. We thought that the release of medial ligament was very important. The varus talus could have enough room to return to normal place under this condition with supramalleolar osteotomy, that whether or not the postoperative TT angle could return to normal matters postoperative functional outcomes. Lateral ligament reconstruction also help correct TT angle. The key point of postoperative functional improvement is how much the cartilage remains. End-stage ankle arthritis with global severe cartilage destruction is not proper for this procedure. Restoring the alignment of ankle and foot could change the abnormal distribution of stress in ankle joint and reduce pain.

This study has some limitations. First, the number of cases was small. One of the problems with this patient population is its heterogeneity. So, more studies with large cases and multi center studies are necessary. Second, all patients were compared with themselves preoperatively and postoperatively. There was no blank control group. Third, the cases were assembled retrospectively in one hospital and may not be representative of other patients undergoing supramalleolar osteotomy owing to differences in ethnicity and socioeconomic status. The generalizability of the findings to different ethnical, racial, and socioeconomic populations is unknown. The strength of this study is that it shows that Takakura 3B ankle arthritis is not a contraindication or relative contraindication for supramalleolar osteotomy.

In conclusion, normal alignment of ankle and hindfoot is very important for varus type ankle osteoarthritis. That whether or not the postoperative TT angle could return to normal matters postoperative functional outcomes. A lot of patients with large preoperative TT angles still achieved good results. Supramalleolar osteotomy can restore the malalignment of ankle joint and modify the abnormal stress distribution so as to achieve functional improvement and improve radiographic stages. Osteotomy for 3B ankle arthritis can have positive outcomes and delay the time of ankle replacement or arthrodesis.

## Figures and Tables

**Table 1:** Summarized demographic data.

parameter	Data
Number of ankles, n	21
Male:female, n(%)	3(14.3%):18(85.7%)
Side (left:right), n(%)	8(38.1%):13(61.9%)
Age at surgery	53.7±5.8(39-61y)
Postoperative Takakura stage, n(%)	
Stage 1	2(9.5%)
Stage 2	11(52.4%)
Stage 3A	4(19.0%)
Stage 3B	4(19.0%)

**Table 2:** Radiographic and functional outcomes.

Parameter	preop	postop	t	p
TAS, deg	82.8±2.4	90.3±2.3	11.1	<0.001
TLS, deg	77.5±2.8	79.4±2.7	3.18	0.0047
TT, deg	13.4±3.6	4.8±3.6	9.78	<0.001
AOFAS-AH	48.0±15.8	74.8±11.5	6.55	<0.001
VAS	5.7±1.3	2.5±1.9	6.37	<0.001
SF-36	41.2±13.1	66.7±14.9	6.28	<0.001
AOS	61.4±12.5	27.5±17.8	7.23	<0.001

## Data Availability

All data included in this study are available in the Supplementary Material.

## Supplementary Materials

Supplementary Materials The supplementary material contains detailed patients' information.
