# Supplementary material for: Medial Open-Wedge Supramalleolar Osteotomy for Patients with Takakura 3B Ankle Osteoarthritis: A Mid- to Long-Term Study
Source: Biomed Res Int. 2019 Jun 17;2019:7630868. doi: 10.1155/2019/7630868 (PMC6601490; doi:10.1155/2019/7630868)
Supplement: Supplementary Materials — The supplementary material contains detailed patients' information. [file 7630868.f1.docx]

Detailed patients’ information

| No. | age | gender | side | BMI | duration of symptoms,years | ankle sprain history | follow-up time,months | calcaneal osteotomy | lateral ligament reconstruction | preoperative TT angle,deg | postoperative TT angle,deg | preoperative VAS score | postoperative VAS score |
| --- | --- | --- | --- | --- | --- | --- | --- | --- | --- | --- | --- | --- | --- |
| 1 | 46 | F | L | 25.8 | 20 | Y | 63 |  | Y | 11.5 | 1.9 | 9 | 0 |
| 2 | 58 | F | R | 31.2 | 20 | Y | 67 |  |  | 11.4 | 8.6 | 4 | 4 |
| 3 | 55 | M | R | 24.8 | 38 | Y | 106 |  | Y | 15.0 | 1.9 | 4 | 0 |
| 4 | 56 | F | R | 24.5 | 10 | Y | 106 |  | Y | 9.4 | 1.9 | 8 | 2 |
| 5 | 54 | F | R | 24.2 | 30 | Y | 109(TAR) |  | Y | 14.7 | 8.5 | 6 | 6 |
| 6 | 39 | F | R | 18.3 | 2 | Y | 110 |  |  | 7.6 | 1.5 | 5 | 0 |
| 7 | 50 | F | L | 29.1 | 15 | Y | 61 | Y |  | 12.4 | 11.5 | 6 | 5 |
| 8 | 47 | F | L | 21.5 | 40 | Y | 70 | Y |  | 9.0 | 5.4 | 5 | 4 |
| 9 | 50 | F | L | 23.9 | 10 | Y | 113 | Y |  | 19.2 | 8.2 | 6 | 3 |
| 10 | 60 | F | L | 25.9 | 30 | Y | 86 | Y |  | 14.8 | 1.0 | 6 | 4 |
| 11 | 58 | F | R | 21.2 | 18 | Y | 69 | Y | Y | 18.4 | 12.5 | 5 | 5 |
| 12 | 58 | F | R | 24.0 | 25 |  | 72 | Y |  | 9.2 | 1.4 | 7 | 3 |
| 13 | 61 | M | R | 25.7 | 2 | Y | 96 | Y | Y | 13.2 | 7.9 | 5 | 4 |
| 14 | 60 | F | R | 25.8 | 40 | Y | 107 | Y | Y | 12.3 | 4.2 | 6 | 1 |
| 15 | 50 | F | R | 25.1 | 2 |  | 83 |  |  | 11.0 | 2.8 | 4 | 1 |
| 16 | 57 | F | L | 23.1 | 20 | Y | 70 |  |  | 20.4 | 2.9 | 7 | 3 |
| 17 | 50 | M | L | 33.1 | 20 | Y | 98 |  | Y | 12.8 | 2.9 | 4 | 0 |
| 18 | 48 | F | R | 23.0 | 2 | Y | 79 | Y | Y | 18.7 | 7.2 | 5 | 2 |
| 19 | 61 | F | R | 23.8 | 8 |  | 75 |  | Y | 15.7 | 5.8 | 6 | 3 |
| 20 | 52 | F | L | 25.2 | 40 | Y | 125 |  | Y | 14.6 | 0.2 | 6 | 0 |
| 21 | 57 | F | R | 24.9 | 3 | Y | 76 |  | Y | 10.6 | 2.0 | 5 | 2 |
